# Dissecting the Role of SMYD2 and Its Inhibitor (LLY-507) in the Treatment of Chemically Induced Non-Small Cell Lung Cancer (NSCLC) by Using Fe_3_O_4_ Nanoparticles Drug Delivery System

**DOI:** 10.3390/ph16070986

**Published:** 2023-07-10

**Authors:** Aasma Munawwar, Amna Sajjad, Azhar Rasul, Mehran Sattar, Farhat Jabeen

**Affiliations:** Department of Zoology, Government College University Faisalabad, Faisalabad 38000, Pakistan; asmashahid787@gmail.com (A.M.); azharrasul@gcuf.edu.pk (A.R.); mehran6566.mm@gmail.com (M.S.); farhatjabeen@gcuf.edu.pk (F.J.)

**Keywords:** SMYD2, LLY-507, IONPs, adenocarcinoma, NSCLC

## Abstract

Cancer therapies based on nanoparticles with a loaded drug can overcome the problem of the drug’s toxic effects in the traditional chemotherapeutic approach. In this study, we loaded LLY-507, a potent inhibitor of SMYD2, a methyltransferase enzyme, on iron oxide nanoparticles (IONPs). The prepared nanoparticles were characterized by microscopic analysis, loading efficiency, and drug release studies. Microscopic examination revealed an average grain size of 44 nm. The in vitro effect of LLY-507-IONPs, LLY-507, and IONPs was determined by MTT analysis (A549 cells) and hemolysis studies. IONPs have almost negative hemolytic activity in blood. The cell viability assay revealed IC50 values of both LLY-507 alone and LLY-507-loaded IONPs against A549; the lower value of the drug loaded on NPs (0.71 µg/mL alone and 0.53 µg/mL loaded on NPs) shows strong synergistic anticancer potential. We further tested the role of loaded NPs in a urethane-induced lung cancer mouse model (n = 40 mice in three independent trials, 20 mice in control group) to check the role of SMYD2 at various time points of lung cancer development. The loss of SMYD2 due to LLY-507 suppressed tumor growth, emphysema, hemorrhage, and congestion considerably. Hence, it can be concluded that the SMYD2 inhibitor has an anti-inflammatory effect on the mouse lung and suppresses tumor growth by inhibiting the SMYD2 protein.

## 1. Introduction

Lung cancer incidence and deaths stand at first position worldwide due to a lack of early diagnosis and late-stage presentation [1]. According to global cancer statistics, 19.3 million new cancer cases with 10.0 million cases of cancer-related mortality were reported worldwide in 2020 [2].

Chemotherapy, immunotherapy, radiation therapy, and surgery are the four primary therapeutic modalities employed worldwide for the treatment of cancer [3]. Among the major limitations of these treatment methods, one of the fundamental issues is the cellular toxicity induced by the non-targeted delivery of these agents [4]. Hence, cancer treatment is often terminated due to the immense side effects of chemotherapeutic drugs such as immunity reduction and bone marrow depression [5].

Over the past few years, nanoparticle administration in cancer therapy has gained much attention. Nanoparticles, polymeric microparticles, niosomes, and liposomes with the appropriate physiochemical and biopharmaceutical properties can target the affected tissue and release a loaded chemotherapeutic drug [6]. The significant carrier characteristics of nanoparticles (NPs) have increased their usage in cancer therapies [7]. Iron oxide nanoparticles (IONPs) have attained particular interest in pharmaceutical research as well as biomedical applications due to their biocompatibility, low toxicity, stability, physical properties, and availability of surface functionalization. Moreover, IONPs can release a drug at the targeted site with the use of an external magnetic field [8,9,10]

A variety of nanoparticles are used to induce cytotoxicity in cancer cells. The idea behind the usage of nanoparticles involves the production of reactive oxygen species (ROS) which results in apoptosis and necrosis of cancer cells. The combined effect of IONPs and the anticancer drug doxorubicin has been tested and is somewhat different from conventional doxorubicin therapy as the combined effect of both has great effectiveness against tumors [11,12,13]. Primary and distal lung cancer growth was inhibited by using cisplatin-loaded gold nanoshells to mediate chemo-photothermal therapy (CDDP-loaded HCP@GNSs), and those nanocarriers had high biocompatibility, strong cytotoxicity against A549 cells, and anticancer activity in an in vivo xerograph model [14]. In the same way, IONPs have shown further advantages over other nanoparticles, including prolonged blood circulation retention duration, quick clearance, minimal adverse effects, superior imaging, and therapeutic efficacy [15]. Without any surface coating, IONPs are unable to load sufficient drugs [16]. Therefore, different kinds of hydrophilic and biocompatible polymers, including dextran [17], polysaccharides [18], poly-ethylene glycol (PEG) [19], poly-aspartate [20], and polyvinyl alcohol (PVA) [21], are coated on IONPs to make their surfaces functionalized for drug loading. Nanoparticles are much more stable with the coated PVA, making these nanoparticles a more efficient material for cancer drug delivery [13,22].

SMYD2 is a methyltransferase enzyme, a member of the SMYD family, identified as an oncogene. It is a SET and MYND domain-containing protein 2 that methylates histone and non-histone proteins associated with active transcription and is involved in the progression of several malignancies [23], including non-small cell lung carcinoma, colon cancer [24], triple-negative breast cancer [25], hepatocellular malignancy [26], and cervical cancer [27]. SMYD2 plays a vital role in cancer development by enhancing the activity of PARP1 (oncogenic protein) [28] and inhibits the activity of Rb [29], p53 [30], and PTEN (tumor suppressor proteins) [31]. SMYD2 is involved in many different cancers, but its role in NSCLC is not yet completely elucidated. There are different kinds of SMYD2 inhibitors available, such as AZ505 [32] A-893 [33], BAY-598 [34], and LLY-507 [35]. Among them, LLY-507 (3-cyano-5-{2-[4-[2-(3-methylindol-1-yl)ethyl]piperazin-1-yl]-phenyl}-N-[(3-pyrrolidin-1-yl)-propyl]benzamide) is preferred due to its biophysical, biochemical, and cellular characteristics. LLY-507 has a low IC50 (in the nanomolar range) in SMYD2-related biochemical assays, and it is highly selective for SMYD2 compared to 24 other related proteins [36]. For cancers in which SMYD2 is overexpressed, LLY-507 has shown antiproliferative activity [35].

To study the basic lung tumor biology and the effect of different drugs for suppressing tumor growth, the urethane lung cancer model is used in different studies [37]. In a related study, AZ505, another strong SMYD2 inhibitor, blocked SMYD2 and significantly reduced cancer cell proliferation, migration, and invasion in an in vitro and in vivo xenograft mice model [38]. Urethane is inexpensive and attractive in lung tumor induction, as it is relatively safe to handle and water-soluble. For this purpose, tumorigenesis was induced by intraperitoneal injections in 8- to 10-week-old mice [39]. Further research revealed that cisplatin-resistant NSCLC cells expressed and activated SMYD2 at higher levels. On a molecular level, SMYD2 promotes cell motility, expands the tumor sphere, and inhibits apoptosis, which is reliant on p53K370 methylation. In vitro and in vivo cisplatin sensitivity would increase with the inhibition or knockdown of SMYD2. Angiogenesis in colorectal cancer (CRC) was previously studied using the BAY-598 inhibitor in a nude BALB/C mouse xenograft model, and it was found to be significantly reduced [40].

In this study, we loaded LLY-507 on IONPs as a lung cancer treatment to reduce toxicity in vitro and in vivo; in this way, we can prevent massive drug loss by targeting the diseased organ. SMYD2 has a higher expression in multiple malignancies, including lung cancer, and is responsible for the worst outcomes and poor prognosis. Regarding current treatments, therapies are non-specific to cancer cells. So, specific drug administration is required to avoid cytotoxic effects in the body. The novelty of this work is the reduction in tumor growth in mouse lungs by inhibiting SMYD2 by using LLY-507-loaded iron oxide nanoparticles in the urethane-induced lung cancer mouse model.

## 2. Results

### 2.1. Characterization of Uncoated IONPs

The characterization of IONPs by atomic force microscopy (AFM), scanning electron microscopy (SEM), and energy-dispersive X-ray analysis (EDX) is presented in the supplementary data.

### 2.2. Magnetic Analysis

**Figure 1:**
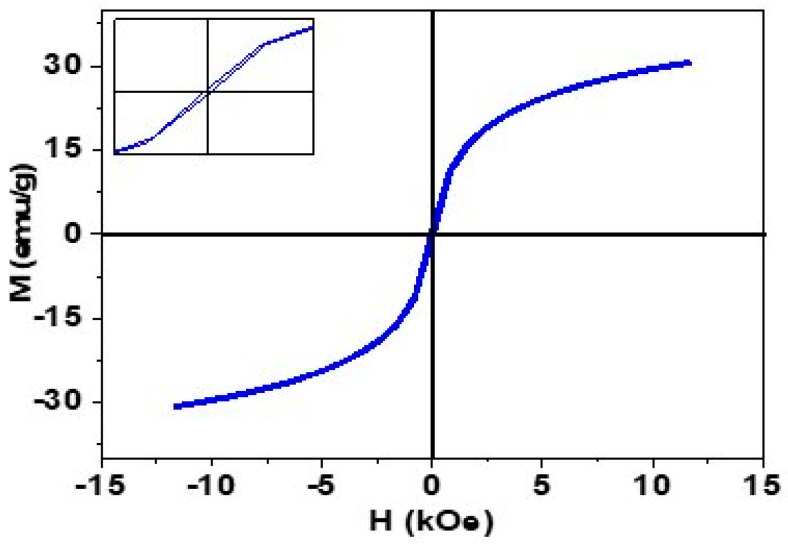
Magnetization curve (M-H) curve of synthesized IONPs.

Vibrating sample magnetometer (VSM) analysis was performed to investigate the magnetic properties of synthesized IONPs (shown in Figure 1. The VSM curve confirms the superparamagnetic nature of IONPs and saturation magnetization of 30.62 emu/g with negligible hysteresis (inset graph).

This superparamagnetic nature is particularly important in drug delivery applications because these particles do not experience any kind of intrinsic magnetic force in the absence of an external magnetic field [41,42].

### 2.3. FTIR Analysis

**Figure 2:**
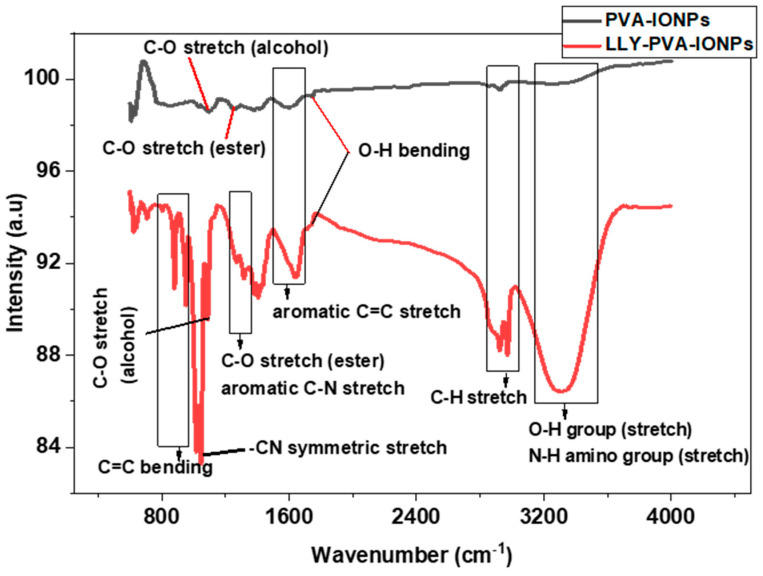
FTIR spectra of PVA-IONPs and LLY-507-loaded PVA-IONPs.

FTIR analysis was carried out to see the composition of PVA-IONPs and drug-loaded PVA-IONPs in powder form using air as a background (Figure 2). The peak analysis also showed the interaction of the drug with PVA-IONPs. Characteristic peaks of PVA that appeared at different wavenumbers are assigned to OH, CH, C=C, and CO stretching. For drug-loaded IONPs, these peaks gain in intensity, and additional peaks appear for the NH amine group, aromatic CN stretching, and symmetric –CN stretching from the piperazine moiety from the drug. A shift in wavenumber occurs for CH, C=C, and CO stretching in the drug-loaded particles. The presence of new signals from the drug and the shift in wavenumbers prove that the drug has been attached to the particles. Similar evidence of drug attachment is also available in the literature.

### 2.4. Drug Loading Analysis

**Figure 3:**
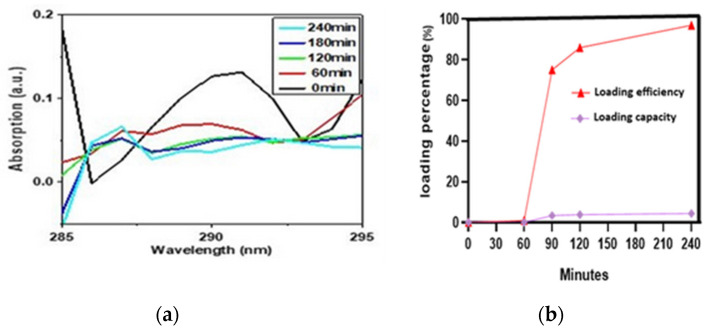
(**a**) UV-Vis absorption spectra for LLY-507 at 0, 60, 90, 120, and 240 min during loading on IONPs. (**b**) Loading efficiency and loading capacity at different time intervals (0, 60, 90, 120, and 240).

For UV-Vis analysis, we took 6.9 mg IONPs in 50 mL DMSO and added 0.3 mg drug into the beaker containing the IONPs. We took the UV spectrum of the supernatant just when the drug was added. The curve at 0 min showed the spectrum of pure drug with maximum absorption at 289 nm wavelength representing a significant amount of the drug in the supernatant. After that, the spectrum was taken at different time intervals to see how much drug had been loaded into the particles. As expected, the absorption decreased with time because of the low availability of the drug in the supernatant, indicating the effective loading of the drug on the IONPs at the bottom of the beaker. At 240 min, the minimum absorption intensity of 0. 003a.u reflected the minimum availability of the drug in the supernatant and hence maximum loading on IONPs. (Figure 3a)

It can be seen in (Figure 3b) that as time increases, the loading capacity (LC) and loading efficiency (LE) of PVA-IONPs for LLY-507 also increase. The loading capacity and efficiency percentage for LLY-507 were very low (1% and 0.046%, respectively) at 60 min. At 240 min, the highest loading efficiency of 97% (970 mg/g) and loading capacity of 4.4% (44 mg/g) were achieved. Since we had a low quantity of the drug available for the experiment (only 0.3 mg to load on 9 mg IONPs) due to its high cost, the capacity is low, but the efficiency is high because drug capacity actually refers to the mass of loaded drug in the total mass of nanoparticles.

### 2.5. Hemolysis Activity

**Figure 4:**
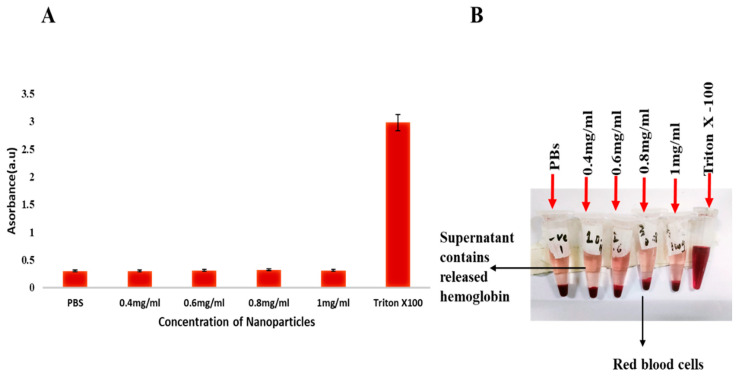
(**A**,**B**) The graph shows the comparison of hemolysis activity of Triton X-100, PBS, and iron oxide nanoparticles at different concentrations.

The PVA-coated IONPs showed negligible hemolysis as compared to negative and positive controls. These nanoparticles showed remarkable biocompatibility in human blood cells, as their hemolysis percentage (less than 5%) was almost equal to or near that of PBS (*p* < 0.05) (negative control, phosphate-buffered saline). While the Triton X-100 value was greater than 5%, all the test sample values were comparable with both the negative and positive controls. These results are favorable for injections, as these nanoparticles have high biocompatibility. So, these nanoparticles are extremely safe for in vivo trials (see Figure 4, a representative photograph showing hemolysis activity using Triton X-100 as a positive control (B); *p* < 0.05, n = 3).

### 2.6. Cell Viability Assay

**Figure 5:**
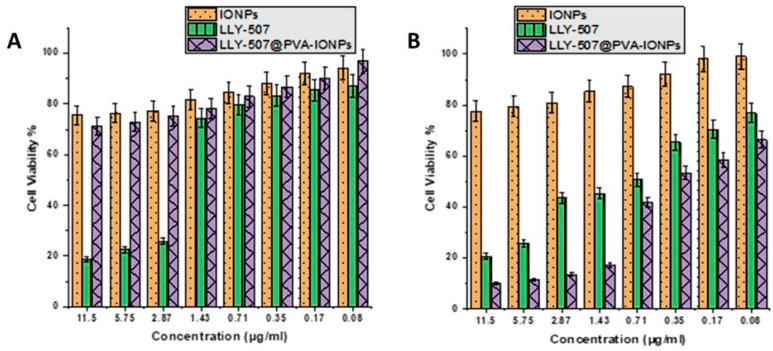
Cell viability percentage of A549 at various concentrations of LLY-507-loaded IONPs from 11.5 µg/mL concentration to 0.08 µg/mL after 48 h (**A**) and 72 h (**B**).

The cell viability was assessed using the MTT assay. The cell viability test of the methyltransferase inhibitor LLY-507 alone and LLY-507 loaded on PVA-IONPs was carried out on the A549 cell line. The cytotoxicity of the drug alone and drug-loaded NPs was evaluated at various concentrations (11.5 µg/mL to 0.08 µg/mL) for 72 h. LLY-507 exhibits an inhibitory effect against A549 cells in a dose-dependent manner. At 48 h, the IC50 of LLY-507 alone was 2.13 µg/mL against A549 cells (Figure 5A). At 72 h, a prominent inhibitory effect was noticed for LLY-507 against A549 cells in a dose-dependent manner (Figure 5B). The IC_50_ of LLY-507 after 72 h was 0.71 µg/mL, which is much lower than the IC50 of LLY-507 at 48 h; this might be due to the antiproliferative effect of LLY-507 against cancer cells decreasing the overall cell number and the requirement to kill the 50% of the population. The IC_50_ of LLY-507-loaded IONPs used against A549 after 72 h was 0.5 µg/mL. Cell viability increased gradually, and a sudden increase in viable cells was observed after the IC50 of LLY-507-loaded IONPs and LLY-507 alone (n = 3 and *p*-value < 0.05).

### 2.7. Lung Cancer Mouse Model Development and Its Treatment

**Figure 6:**
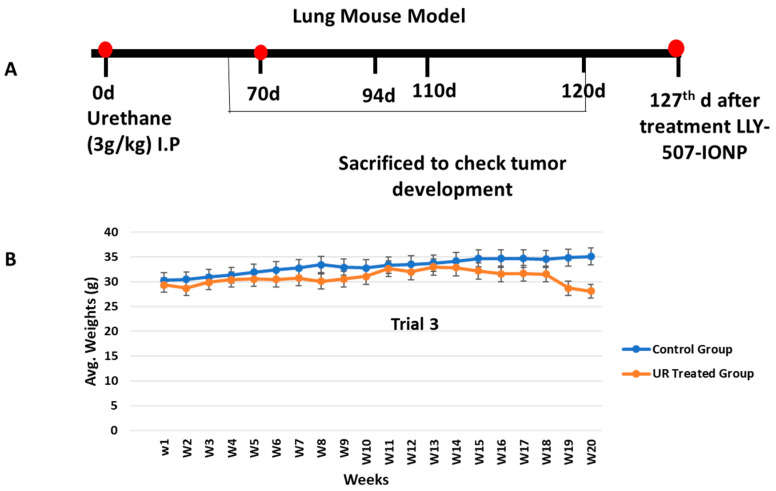
(**A**) Schematic representative figure showing the development of the urethane-induced lung cancer mouse model. (**B**) The experiment was repeated 3 times. Weekly weights were measured to see the difference between the control and urethane groups.

**Figure 7:**
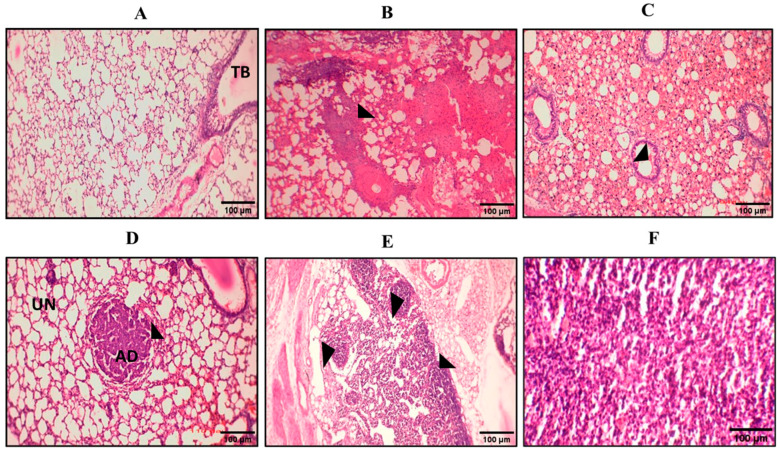
Representative pictures of hematoxylin and eosin (H&E) staining at various stages. Here we can see pictures of H&E staining from (**A**–**F**). (**A**) The control group was normal without any change; TB = terminal bronchi. (**B**) On the 70th day, glandular proliferation started. (**C**) On the 94th day, the glands began to unite with each other. (**D**) Adenoma formation on the 110th day; UN = uninvolved area, AD = adenoma. (**E**) Various adenomas started to proliferate and became united on the 120th day. (**F**) Adenocarcinoma.

We repeated our urethane-induced trial three times to investigate the effect of our drug-loaded IONPs on the lungs, kidney, liver, spleen, and heart. We administered a high dose of urethane (3 g/kg, which does not produce massive mortality in mice) in BALB-c mice. For the development of tumors in the lungs at different checkpoints, we sacrificed animals (at the 60th, 70th, 94th, 110th, and 120th days of trial for the assessment of tumor progression in mice). There was no change in the control group; in the lung cancer mouse model, inflammation and hemorrhage were seen on day 60 (n = 1), and congestion of blood vessels was present on the 70th day (n = 1). On the 94th (n = 2) and 110th days, individual rounded glands were formed and started to proliferate (adenoma formation). Weekly weights were noted throughout the trial (Figure 6). The individual glands started to unite with each other (See Figure 7). 

On the 120th day, different treatment groups were selected. We used LLY-507, a potent SMYD2 inhibitor, to dissect the role of SMYD2 in non-small lung cancer in a chemically induced urethane model as it is a valuable and inexpensive tool to generate lung tumors in mice. Briefly, four groups were formed: (A) 0.9% NaCl (positive control, urethane) (n = 5), (B) LLY-507 (n = 4), (C) IONPs (n = 4), (D) LLY-507-loaded IONPs (n = 4). There was no change in the negative control (group A, average weight = 37 g), nor was there a considerable change in the group treated with LLY-507 alone (average weight = 32 g). The IONP group (average weight = 35) had no congestion in the lungs and exhibited minimal inflammation, and the glandular epithelium was disrupted in that group of mouse lungs. However, amazing results were seen in the LLY-507-loaded IONP group (average weight = 37 g), as there was no inflammation or congestion in that group, meaning that the SMYD2 inhibitor LLY-507 has an anti-inflammatory effect in the urethane-induced lung cancer model. It is already known that inflammation along with various factors plays a great role in tumorigenesis. There were no considerable changes seen in weights after treatment, but we had to sacrifice animals due to health issues induced by urethane such as short breathing, allergies, and irritations.

### 2.8. NSCLC Markers in Urethane Lung Cancer Mouse Model

**Figure 8:**
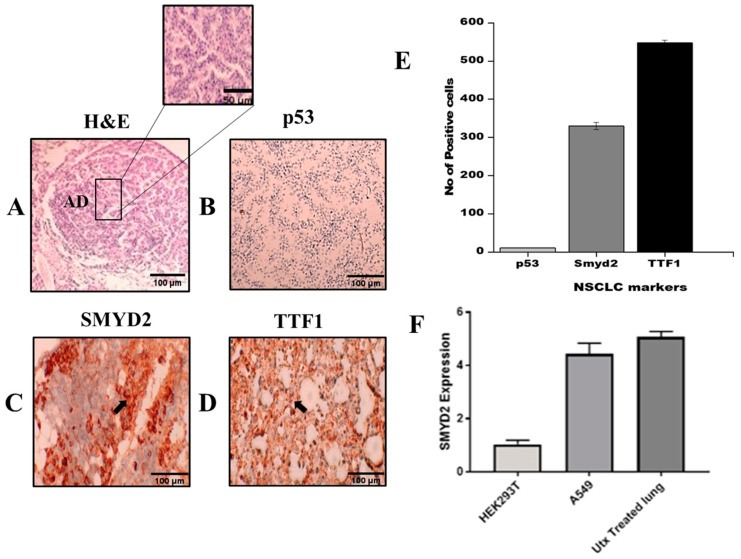
(**A**) Representative picture of histology of adenocarcinoma exhibits glandular origin. (**B**) Downregulation of p53 expression, due to repressive activity of SMYD2. (**C**) Positive expression of SMYD2 correlated with p53 suppression. (**D**) TTF1 expression validates NSCLC development. (**E**) Quantification of NSCLC markers by counting cells positive for p53, SMYD2, and TTF1. (**F**) RT-qPCR was used to detect the relative expression of SMYD2. A high expression level of SMYD2 was found in urethane-treated lungs. The data were calculated in triplicate (n = 3 and *p*-value < 0.05). UTX = urethane.

TTF1 (thyroid transcription factor 1), which is a commercially known marker for a subtype of NSCLC (adenocarcinoma) and had shown positive expression in mouse lungs, was highly positive in urethane-induced NSCLC (see Figure 8D), while p53 expression was negative in our urethane-induced lung cancer model (see Figure 8B). High SMYD2 expression can be seen in the in vivo model, which corresponds to NSCLC literature (see Figure 8C). The number of TTF1-positive cells (10 microscopic pictures were selected randomly on each slide, and positive cells were counted) was higher than the number of SMYD2-positive cells, and p53-positive cells were fewer in number (Figure 8E). RT-qPCR analysis showed that SMYD2 expression is higher in A549 cells and urethane-treated mice (UTX) than in HEK293T cells. To validate in silico analysis results, RT-qPCR was performed on cDNA, obtained from unhealthy and normal tissue samples. As shown by the results in Figure 8F, the expression of SMYD2 in lung cancer samples had significantly increased relative to the control.

### 2.9. p53 Regulation in a Urethane-Induced Lung Cancer Mouse Model

**Figure 9:**
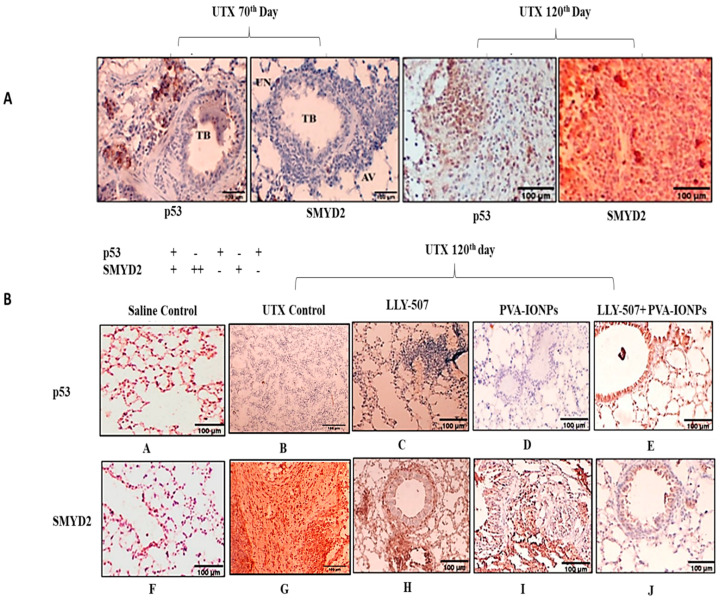
(**A**) Immunohistochemistry of urethane-induced lung cancer model on 70th and 120th days. (**B**) LLY-507 significantly inhibited SMYD2 expression in tissues while p53 expression was restored (**A**–**J**).

SMYD2 is a methyltransferase enzyme that is known to inhibit p53-mediated apoptosis via methylation upon DNA impairment [43]. We checked the expression of SMYD2 and p53 in parallel in the urethane lung cancer mouse model at different time periods of carcinoma development as well as after the treatment with IONPs loaded with the potent SMYD2 inhibitor LLY-507. The SMYD2 expression was negative in the initial period of the trial (60th and 70th days), while the p53 expression was still present. (Figure 9A)

On the 120th day, SMYD2 expression was at its peak, and p53 expression was moderately high, which correlates with the p53 expression being suppressed by SMYD2-mediated methylation. (Figure 9A)

The inhibition of SMYD2 by LLY-507-loaded IONPs decreased the growth of tumors in mouse lungs, and expression of SMYD2 also decreased, which corresponds to p53 upregulation in LLY-507-treated mouse lungs. LLY-507 alone or IONPs have less significant results on urethane-treated lungs and have minimal impact on inflammation or reduction in emphysema. However, in the case of LLY-507-loaded IONPs, cell proliferation was reduced, and the emphysema condition was controlled. This is all because of SMYD2 downregulation and p53 upregulation in the lungs by the inhibitor. These findings suggest that LLY-507-loaded IONPs along with any other commercial drug will produce a pronounced effect in the treatment of non-small cell lung cancer. (Figure 9(A–J))

## 3. Discussion

In this study, the characterization of coated particles via atomic force microscopy (AFM) and energy-dispersive X-ray spectroscopy (EDS) showed that iron (Fe) and oxygen (O) are the basic elements of IONPs, and the average grain size is 44 nm with non-uniform distribution, as given in the supplementary information (S1). It is publicized that 50 nm is the normal optimal size of nanoparticles for retention in cancer cells as compared to smaller sizes of 20 nm or larger sizes such as 200 nm. This is because smaller-size nanoconjugates clear quickly from the body and larger NPs face difficulty penetrating the tumor tissues [44]. The functionalization of PVA coating not only stops clumping but also affects the growth of the IONPs [45].

Furthermore, the VSM curve has confirmed the superparamagnetic nature of IONPs, which is particularly important in drug delivery ability due to the absence of any intrinsic magnetic force [41,42].

The overall loading efficiency of PVA-IONPs for LLY-507 is 95%, while the loading capacity is 4.2%, as calculated from the relative absorbance of LLY-507 with UV-Vis spectroscopy. The low value of the loading capacity is due to the fact that a very low quantity of the highly potent drug (LLY-507) was used for loading purposes, and the amount of available drug relative to the surface area of the IONPs is low. In a recent study, ultrasound-induced DOX release from gold nanoparticles was predicted using a computational model of LIPUS-triggered DOX release combined with DLVO theory. In an ex vivo tissue model, LIPUS-induced DOX release from GNP drug carriers was accomplished [46]. 

Hemolysis is one of the most important and simple tests to check the biocompatibility of any nanocomposite in blood. It is a good tool before the administration of any intravenous injection for in vivo analysis as hemolysis can lead to anemia, jaundice, and other serious effects on health [47]. A hemolysis rate of less than 5% is considered good for safe in vivo experiments [48], which is in line with our studies.

A chemically induced lung cancer model is a valuable tool for experimental oncology. Lung carcinogenesis induced by urethane has been used for the study of lung cancer risk and Kras-driven tumor progression, investigation of the antiproliferative effects of drugs, tumor biology, and early detection of cancer [39]. The weight of mice had been noted from day 1 to the end of the trial, and an average 10 to 12% reduction in weight of urethane-treated mice was observed as compared to the control group, as mentioned in previous literature [49].

Normally, inflammation caused by the innate immune response is critically important for host defense, clearance of damaged or mutated cells, and managing tissue homeostasis. However, abnormal inflammation promotes the proliferation and invasion of cancer cells through the amplification of enzymes that produce tissue damage, induce reactive oxygen species that add further mutations, favor angiogenic factors and cytokines, and maintain the survival of cancer cells [50]. Treatment with LLY-507-loaded PVA-IONPs has showed in a significant decrease in inflammation in mice lungs. As it is known, LLY-507 has anti-inflammatory property and this is proved in our results by reducing inflammation, so it could produce better results in the treatment of NSCLC along with any commercially available drug to produce a pronounced effect.

According to a prior study, LLY-507 causes apoptosis and inhibits HGSOC cell proliferation [23]. In a previous study, breast cancer cells were 5 times more sensitive to LLY-507 after seven days of treatment compared to three to four days, indicating that at least some of the SMYD2-dependent epigenetic mechanism may be responsible for the proliferation of breast cancer cells. Liver cancer cells’ sensitivity to LLY-507 did not increase in a time-dependent manner [35]. The growth of cell lines with ovarian clear cell carcinoma is inhibited by SMYD2 suppression. LLY-507, a selective SMYD2 inhibitor, was applied to OVTOKO, TOV21-G, and OVMANA cells in doses ranging from 0.001 to 10 M [51]. In ovarian cancer, the IC50 of LLY-507 ranges between 1.77 μM and 2.90 μM, and it is normalized in the control (DMSO) [23]. In the present study, LLY-507 was loaded on PVA-coated IONPs to evaluate its cytotoxicity on A549 cells (with a concentration range of 11.5 µg/mL to 0.08 µg/mL) in a dose-dependent manner. We used drug-loaded nanoparticles in mice to suppress the expression of SMYD2 and reduce tumor growth as it has already been reported that SMYD2 promotes lung tumorigenesis. The inhibition of SMYD2 could enhance p53 pathway activity and induce cell apoptosis by regulating its target genes, including p21, GADD45, and Bax [52].

Thyroid transcription factor 1 (TTF-1) is a well-known marker for adenocarcinoma. The estimated sensitivity ranges from 70 to 96 percent [53]. In our study, mouse lungs showed a high TTF1 expression. p53 is a tumor suppressor gene that plays a crucial role in various cellular functions, including autophagy, apoptosis, senescence, and the metabolic reprogramming process [54]. Mutation in the p53 transcription factor through either direct mutation or abnormality in one of its regulatory pathways is a hallmark of many tumors. Mutated TP53 occurs in almost 34% of NSCLC patients [55,56]. In our study, p53 expression was initially suppressed by SMYD2 overexpression; later, with loaded drug delivery, p53 expression was restored. SMYD2, a methyltransferase enzyme, modifies gene transcription and signaling pathways and thus is involved in tumor progression [52].

This study could be useful for researchers working in the field of drug delivery and the in vivo use of nanoparticles loaded with inhibitors, allowing drug loss and cytotoxic effects to be reduced.

## 4. Material and Methods

### 4.1. Physiochemical Characterization of IONPs

Atomic force microscopic (AFM) analysis was carried out using Nano-Solver, NT-MDT, in semi-contact mode to study the microstructure and surface morphology of IONPs. SEM analysis was also performed to see the morphology and grain size of the nanoparticles by using field-enhanced SEM (Model: Nova NanoSEM 450, high vacuum mode, 10 kV). EDX was performed using the same instrument for compositional analysis (supplementary data). The magnetic nature of the synthesized nanoparticles was studied using a vibrating sample magnetometer (Model: Lakeshore’s 7407 vibrating sample magnetometer).

For compositional analysis of PVA-IONPs and to see the bonding of the drug onto PVA-IONPs, Fourier transform infrared (FTIR) spectra were taken in transmission mode using a Shimadzu IR Prestige-21 (4000–500 cm^−1^ wavenumber range). The scans were taken at 4 cm^−1^ resolutions to record both spectra while taking air as the background.

### 4.2. Synthesis of Iron Oxide NPs

The synthesis of iron oxide NPs was carried out using the co-precipitation method [57]. A combined solution of ferrous chloride and ferric chloride was prepared in 100 mL of deionized water by dissolving 0.27 M ferric chloride and 0.15 M ferrous chloride. Instead of using the standard ratio (1:2) of iron II and iron III, we used a 1:1.8 ratio to prepare iron oxide NPs. This concentration ratio was used to maintain the normal 1:2 ratio for iron II and iron III because Fe^2+^ converts to Fe^3+^ in the air or an oxidizing environment. The mixture of iron II and iron III was heated at 80 °C at 800 rpm for 3 h. After that, this solution was poured dropwise into 200 mL of a 0.1 M NaOH solution with constant stirring. Every drop of poured solution was converted to a black precipitate of iron oxide NPs upon reaction with the NaOH solution. These black precipitates of iron oxide NPs were washed three times with deionized water and once with isopropyl alcohol and dried on a vacuum hotplate at 65 °C [58].

The following equations show ferric and ferrous chloride conversion into iron oxide nanoparticles:

(a) FeCl_2_ + 2NaOH → Fe(OH)_2_ + 2NaCl
  Fe(OH)_2_ → FeO + H_2_O
(b) 2FeCl_3_ + 6NaOH → 2Fe(OH)_3_ + 6NaCl
  2Fe(OH)_3_ → Fe_2_O_3_ + 3H_2_O

### 4.3. PVA Functionalization of IONPs

Using an immobilization method, iron oxide nanoparticles were coated with 3 wt% PVA. Three grams of PVA, one gram of fine powder of iron oxide NPs, and 96 mL of deionized water were used for the PVA coating. PVA and iron oxide NPs were mixed in deionized water at 80 °C with continuous stirring. After complete mixing, the temperature was allowed to decrease. The solution was stirred for 11 h to achieve maximum coating on iron oxide NPs. PVA-functionalized iron oxide NPs were separated with the help of a permanent magnet, washed 4 times with deionized water, and dried at 35 °C on a vacuum hotplate.

### 4.4. LLY-507 Loading on PVA-Coated IONPs

**Figure 10:**
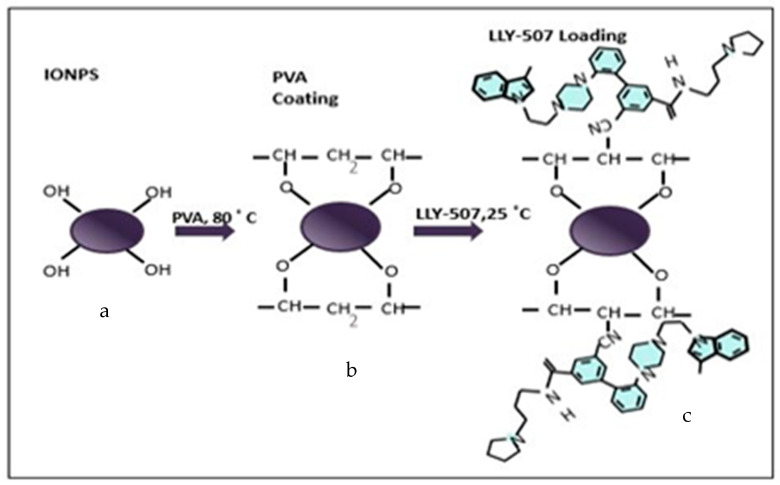
Schematic illustration of methyltransferase inhibitor (LLY-507) loaded on PVA-coated iron oxide NPs. (**a**) Iron II (FeO) and iron III (Fe_3_O_4_) nanoparticles combined, labeled as Fe_3_O_4_. (**b**) Polyvinyl alcohol (PVA) coated on IONPs with the removal of a water molecule on each attachment. (**c**) Methyltransferase (SMYD2) inhibitor, LLY-507, loaded on PVA-functionalized IONPs for anticancer activity.

An anticancer drug, LLY-507 (methyltransferase inhibitor), was loaded on PVA-functionalized iron oxide NPs. The bonding of PVA and LLY-507 with IONPs and PVA, respectively, is schematically shown in Figure 10. The loading of LLY-507 was achieved by dissolving 0.3 mg LLY-507 in 50 mL DMSO followed by the addition of 9 mg PVA-functionalized iron oxide NPs. During the loading process, the solution was kept shaking with an orbital shaker, and drug loading was observed using UV-Vis spectroscopy at different time intervals (0, 60, 90, 120, and 240 min). The absorption of clear supernatant was quantified at varying intervals for this purpose. The typical absorption range for LLY-507 is between 280 and 300 nm.

Using the following equations, the UV-Vis absorption curves were further analyzed to measure the loading parameters, such as loading capacity (LC) and loading efficiency (LE):

$$\mathrm{Loading}\;\mathrm{Capacity}\;\%=\frac{Initial\;Drug-Free\;Drug}{Weight\;of\;Nanoparticles}\;\times 100$$

$$\mathrm{Loading}\;\mathrm{Efficiency}\;\%=\frac{Initial\;Drug-Free\;Drug}{Initial\;Drug}\;\times 100$$

Drug-loaded iron oxide NPs were separated with the help of a permanent magnet and used for further analysis.

### 4.5. In Vitro Hemocompatibility Test 

In hemolysis, the red blood cell membrane is ruptured and hemoglobin is released into the plasma. To check the effects of iron oxide nanoparticles (IONPs) on blood, a hemolysis test was performed. A fresh human blood sample of approximately 5 mL was taken from a volunteer and placed in vacutainer tubes with EDTA to avoid coagulation. The blood sample was centrifuged at 3000 rpm for 20 min, and the plasma was discarded according to safety rules. Blood cells were washed three times with a double volume of phosphate buffer solution (PBS) (pH 7.4). Stock solutions of functionalized PVA-IONPs were made (5 mg/mL), and the serial dilution of test samples was as follows: 0.4 mg/mL, 0.6 mg/mL, 0.8 mg/mL, and 1 mg/mL. For negative control, 1 mL PBS was added to blood cells, and for positive control, 100 μL (4%), Triton X-100 was used. We took 400 μL of blood cells and 1 mL of PBS in each Eppendorf tube, and a 100 μL IONP test sample was added to make the final volume. All samples were incubated at 37 °C for 1 h and then placed in an ice bath for 60 s. Then, samples were again centrifuged at 3000 rpm for 5 min. The supernatant was used to record the absorbance for the quantification of hemoglobin in samples at 540 nm [59].

### 4.6. Cell Viability Assay

The MTT assay is extensively used against various cell lines to estimate the cytotoxicity of any drug or extract. Dulbecco’s Modified Eagle’s Medium, 10% fetal bovine serum, 100 units/mL of penicillin, and 100 g/mL of streptomycin were added to microplates containing human lung adenocarcinoma (A549) cells at 37 °C with 5% CO_2_. The cells treated with DMSO were considered the negative control. Following confluence, the cells were exposed to various doses of LLY-507 alone and LLY-507-loaded IONPs. The microplates were then incubated once again at 37 °C for 4 h after 10 μL (5 mg/mL) of the MTT reagent had been added after 48 h. Finally, DMSO (150 μL) was added to dissolve the formazan crystals, and a Thermo Scientific microplate reader was used to measure the absorbance at 490 nm [60].

### 4.7. Lung Cancer Mouse Model

Adult BALB-c male mice 8–10 weeks old with an average weight of 30 g were taken and kept under the strict guidelines of the National Institute of Health Islamabad. The mice were used in the preparation of a chemically induced lung cancer mouse model.

All the experiments were carried out after the approval of the Ethical Committee for Animal Research Government College University Faisalabad. BALB-c mice were kept in cages according to international standards with an average temperature of 23 °C, a 12 h dark–light cycle, and free access to water and food.

Urethane (ethyl carbamate) (provided by Fluka) was used for the induction of non-small cell lung carcinoma. Urethane was dissolved at 100 mg/mL in 0.9% NaCl. Aliquots of 0.5 mL were made and stored at −20 °C. The two groups of mice named groups A and B were assigned. There were 20 mice in group A (healthy mice) and 20 mice in group B (urethane-treated mice). BALB-c mice were injected with 3 g/kg of urethane with two intraperitoneal injections of 1.5 g/kg within 48 h, and 0.9% NaCl was injected in the control group. It was proven in previous studies that a high dose of urethane does not elicit massive mortality soon after the administration of the drug, and adenomas develop within 48 days. Fully grown tumors were developed after 120 days of urethane induction [61,62] (Figure 7), and after treatment with LLY-507-loaded PVA-IONPs, animals were sacrificed on the 128th day for further analysis. Adenomas are formed in the bronchoalveolar parts of the lungs. So, we took the left lower lobe for snap freezing, and the rest of the whole lung was fixed in formalin for histology and immunohistochemistry

### 4.8. Drug Administration for Treatment

After tumor formation, five groups were made. They were named groups A, B, C, D, and E. Group A comprised healthy mice. Group B consisted of urethane-treated mice; they were given i.v. 0.9% NaCl only. Group C was injected with 30 mg/kg of LLY-507 for three days with an interval of 24 h. In group D, 1 mg PVA-coated iron oxide nanoparticles were dissolved directly in 200 μL PBS and intravenously infused. In group E, mice were i.v. injected with LLY-507-loaded iron oxide nanoparticles (1.5 mg IONPs + 30 mg/kg drug; after loading, loaded IONPs dispersed in PBS). After 7 days of treatment, all the groups of mice were sacrificed, and their organs were harvested for histopathology and immunohistochemistry.

### 4.9. Histology

After fixation in 10% neutral paraformaldehyde solution (PFA) for 12 h or overnight, samples were dehydrated by using different ethyl alcohol grades (50%, 70%, 80%, 90%, 96%) each for two hours except for two changes of 100% alcohol grading (1 h each). Samples were then immersed in butanol overnight but for less than 15 h (all procedures performed in the hood). To clean the samples, two changes were given with paraffin for 4 to 6 h at 65 °C (two times) in the incubator. Then, tissue was embedded carefully in the center of the mold and kept at 4 °C for 7 h, and blocks were stored at room temperature. For the histopathological study, the tissue was sliced into 5 um thick sheets using a microtome. Rehydration of sections was performed with two changes of 100% alcohol, each for two minutes following 96%, 90%, 70%, and 50% for 1 min each. The slides were stained with hematoxylin and eosin. A drop of Entellan was put on the slides, and the slides were covered with coverslips. The slides were observed under a microscope.

### 4.10. Immunohistochemistry

Paraffinized tissue samples were cut into 5 um thick slices. We placed the slides in an oven at 80 °C for half an hour. Then, we passed the slides three times through xylene dip, each for 1 min. We rehydrated the sections with graded alcohol two times for 1 min. Antigen retrieval solution (distilled water + 2 mL retrieval solution (cat# S2367), Agilent, Santa Clara, CA, USA) was used, and the slides were kept in a hot air oven at 97 °C for 45 min. To normalize the slide’s temperature, we kept the slides at RT for 10 min. We washed the slides with distilled water. We encircled the section with a pap pen to avoid wastage of valuable reagents. We placed the slides in a humidity chamber and put a drop of peroxidase-blocking solution 100 μL (cat# S801) on each slide for 30 min. We diluted the primary antibodies rabbit-SMYD2 (1:100, cat# 4251s, cell signaling), TTF1 (cat# 790-4398, Ventana, Tucson, AZ, USA), and mouse anti-p53 (1:50, cat# 2524s, cell signaling) and placed them in a dark place for an hour. Then, we washed the slides with buffer solution and added rabbit anti-mouse secondary antibody (cat# A27025, Thermo Fisher Scientific, Waltham, MA, USA) on the slides, and we incubated the slides for 30 min. We put a few drops of Dab’s chromogen (GV825), Dako, Santa Clara, CA, USA) onto slides and kept them for 20 min. Then, we cleaned the slides with washing buffer. Then, slides were passed through xylene dips and alcohol and counterstained with hematoxylin (Dako manufacturer procedure was followed). For quantification, the number of positive cells was calculated using 10 randomly selected micrograph pictures taken using a microscope (Model HD 1500 T, Meiji techno, Iruma-gun, Japan).

### 4.11. SMYD2 Expression by RT-qPCR

Following a normal approach, total mRNA was extracted from tissue samples using TRIzol (Invitrogen; Thermo Fisher Scientific, Waltham, USA). After RNA samples were quantified and qualified according to manufacturer’s guidelines using a Nanodrop spectrophotometer (Epoch spectrophotometer, BioTek) and standard agarose-gel electrophoresis, respectively, RNA was reverse transcribed into cDNA using the Revert Aid First Strand cDNA Synthesis Kit (Thermo Fisher Scientific, Waltham, USA). RealQ Plus Master Mix Green (Amplicon) and the Applied Biosystems 7500 Real-time PCR instrument were then used to perform RT-qPCR (Thermo Fisher Scientific, Inc., Waltham, MA, USA). To assess the expression levels and standardize the relative expression levels for data analysis, HEK293T was employed as an endogenous control in this stage. Afterward, the data were studied. Each sample was examined in triplicate [63].

### 4.12. Statistical Analysis

Statistical analysis was performed by using IBM SPSS Statistics version 25 for standard deviation and error bars in the groups. Origin 2018 64 Bit was used for graphs.

## 5. Conclusions

This study shows that the SMYD2 inhibitor LLY-507 can be efficiently loaded onto PVA-coated IONPs to overcome the side effects of non-targeted delivery. LLY-507 effectively reduces cancer cell proliferation, specifically in the A549 lung adenocarcinoma cell line as well as in the lung adenocarcinoma mouse model. SMYD2 is an emerging target in cancer research, and its high expression is believed to be involved in poor-prognosis cancers. The urethane-induced lung cancer model in mice is a cost-effective and reliable tool for NSCLC studies. LLY-507 is a potent SMYD2 inhibitor with minimal cytotoxic effects, as confirmed by hemolysis, MTT, and hematological analyses. The novelty of this study lies in treating urethane-induced lung carcinoma using LLY-507-loaded iron oxide nanoparticles to investigate SMYD2’s role in NSCLC treatment. LLY-507-loaded PVA-IONPs significantly inhibited SMYD2 activity, reducing emphysema, congestion, hemorrhage, and tumor growth. Further preclinical investigations are warranted, supported by this research and existing literature. SMYD2 shows promise as a novel target for various carcinomas, particularly in lung cancer, where it contributes to cancer cell proliferation. LLY-507 could be a valuable anticancer drug for inhibiting SMYD2 and reducing proliferation. Magnetically targeted delivery of LLY-507 at a micromolar concentration may enhance its efficacy.

## Data Availability

Data are contained within the article and Supplementary Materials.

## Supplementary Materials

The following supporting information can be downloaded at: https://www.mdpi.com/article/10.3390/ph16070986/s1, Figure S1:( 1a. AFM image of IONPs, (1b. grain size of (nm) IONPs and count; Figure S2: (a) SEM image (b) Corresponding EDS spectra showing the elemental composition of IONPs: Table S1: EDS elemental microanalysis of IONPs.

## Abbreviations

NSCLC (non-small cell lung carcinoma), IONPs (iron oxide nanoparticles), NPs (nanoparticles), PVA (polyvinyl alcohol), SMYD (SET and MYND domain-containing), ROS (reactive oxygen species), PARP1 (poly(ADP-ribose) polymerase 1), Rb (retinoblastoma), p53 (TP53 gene), PTEN (phosphatase and tensin homolog deleted on chromosome 10), LLY-507 (3-cyano-5-{2-[4-[2-(3-methylindol-1-yl)ethyl]piperazin-1-yl]-phenyl}-N-[(3-pyrrolidin-1-yl)-propyl]benzamide), U2OS (osteosarcoma), ESCC (esophageal squamous cell carcinoma), AFM (atomic force microscopy), SEM (scanning electron microscopy), EDX (energy-dispersive X-ray analysis), FTIR (Fourier transform infrared), MTT (microculture tetrazolium test), DMSO (dimethyl sulfoxide), UV-Vis (ultraviolet–visible), EDS (energy-dispersive X-ray spectroscopy).
