# Supplementary material for: Dissecting the Role of SMYD2 and Its Inhibitor (LLY-507) in the Treatment of Chemically Induced Non-Small Cell Lung Cancer (NSCLC) by Using Fe3O4 Nanoparticles Drug Delivery System
Source: Pharmaceuticals (Basel). 2023 Jul 10;16(7):986. doi: 10.3390/ph16070986 (PMC10384399; doi:10.3390/ph16070986)
Supplement: Supplementary file 1 [file pharmaceuticals-16-00986-s001.zip › pharmaceuticals-2336840-supplementary.pdf]

2. Results

Morphology and compositional analysis of uncoated IONPs:

S1. AFM analysis:

Fig. 2 shows the AFM image of synthesized IONPs. AFM image was taken is semi-contact mode. For deposition of IONPs, dispersion of IONPs was made in ethanol and drop casted on glass substrate for AFM imaging. The length and width of cantilever are 135 µm and 35 µm respectively and its thickness is 2 µm. Spherical and agglomerated particles can be seen in the micrograph.

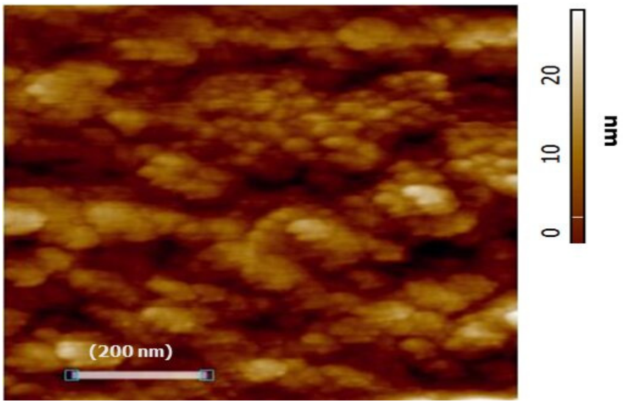

Figure S1a. AFM image of IONPs.

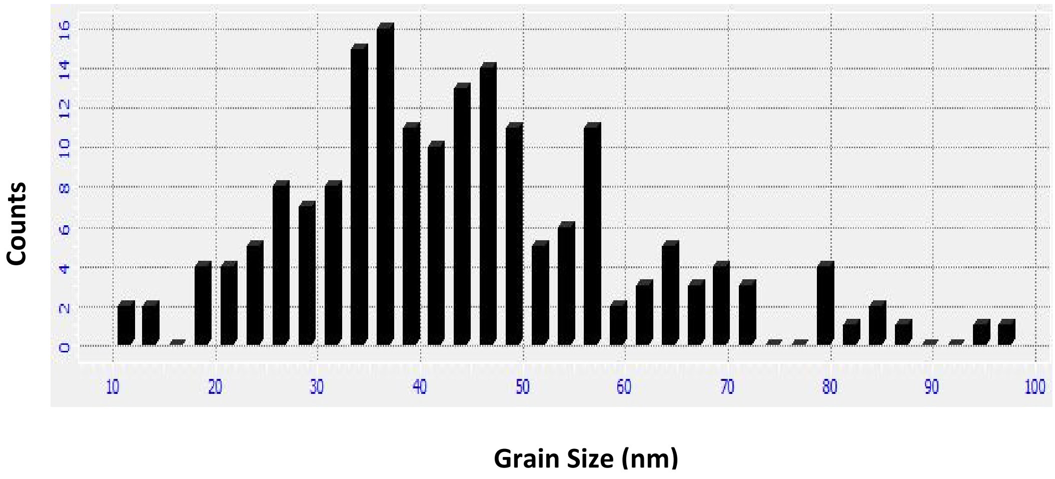

Figure S1b. grain size of (nm) IONPs and count

S2. SEM and EDX Analysis

Fig. 3 shows the SEM image and corresponding EDX spectrum of IONPs. SEM image shows agglomerated particles. The average grain size was measured to be 52 nm using SEM

and AFM images. Energy dispersive spectra (EDS) were used to obtain the elemental configuration or compositional analysis of uncoated IONPs. The EDX spectrum shows the Fe and O peaks elements as constituents of Fe_2O_3 and FeO of the prepared material. The atomic percentage of elements present in the sample is depicted in table 1.

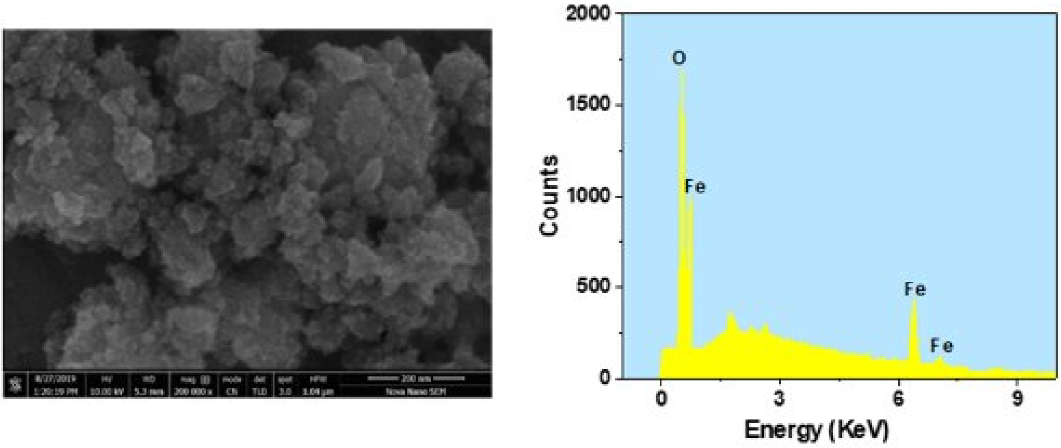

Figure S2. (a) SEM image (b) Corresponding EDS spectra showing the elemental composition of IONPs

Table S1: EDS elemental microanalysis of IONPs

Sr. #	Elements	Wt.%
1	Iron (Fe)	30.53
2	Oxygen (O)	69.46
